# The Effects of the Fear of Missing Out on People's Social Networking Sites Use During the COVID-19 Pandemic: The Mediating Role of Online Relational Closeness and Individuals' Online Communication Attitude

**DOI:** 10.3389/fpsyt.2021.620442

**Published:** 2021-02-18

**Authors:** Francesca Gioia, Giulia Fioravanti, Silvia Casale, Valentina Boursier

**Affiliations:** ^1^Department of Humanities, University of Naples Federico II, Naples, Italy; ^2^Department of Health Sciences, School of Psychology, University of Florence, Florence, Italy

**Keywords:** COVID-19, fear of missing out, online communication attitude, problematic social networking sites use, relational closeness

## Abstract

Forced isolation induced by COVID-19 pandemic dramatically impacted individuals' well-being, reducing the opportunities for social encounters, consequently resulting in a greater use of social media in order to maintain social relationships. Although the range of friend-related activities appeared to be severely constrained during quarantine, the Fear of Missing Out (FoMO) needs to be carefully examined, especially in relation to problematic social networking site use (PSNSU). Indeed, FoMO might enhance individuals' need to stay connected and communicate with other people, leading to PSNSU, in order to face the fear of being invisible in the world of social media in circumstances of physical isolation. The present study sought to evaluate the predictive role of FoMO on PSNSU during the COVID-19 pandemic, testing the mediating effect of online relational closeness and online communication attitude. A total of 487 Italian adults (59.3% women), aged between 18 and 70 years (mean age = 29.85 years; *SD* = 9.76), responded to an online survey during the period of COVID-19 pandemic lockdown in Italy. The survey included self-report measures assessing perceived FoMO, online communication attitude, relational closeness with online friends, and PSNSU. Participants declared they spent significantly more time social networking during the pandemic, particularly women. The total model accounted for a significant amount of variance in participants' PSNSU [*R*^2^ = 0.54; *F*_(9, 447)_ = 58.285, *p* < 0.001). Despite the other people's social rewarding experiences had been drastically reduced by the lockdown, findings showed a direct effect of FoMO on PSNSU. Moreover, FoMO had an effect on online communication attitude and online relational closeness, although only online communication attitude predicted, in turn, PSNSU. Conversely, relational closeness on social networking sites did not predict PSNSU. The present study suggests that, during COVID-19 lockdown, FoMO levels may have strengthened attitudes toward online communication, which, in turn, may have put some individuals at risk of PSNSU.

## Introduction

The impact of the COVID-19 pandemic on people's lives represents a critical issue that deserves empirical examination for mental health science (1). Indeed, the experience of isolation and separateness due to the forced physical-distancing has impacted on people's relationships and well-being, resulting in negative psychological outcomes (2–4), sometimes leading to fatal events (5–7).

In this context, the relevance that fears had on individual behavior and functioning represents an important matter of the debate. Accordingly, an integrated model of understanding fear experiences during the COVID-19 pandemic has been recently proposed, together with a multidimensional assessment for COVID-19-related fears (8, 9). Moreover, the experience of fear specifically related to interpersonal features (i.e., the fear of missing out and fear of not mattering to other people), resulting from individuals' psychological needs not met due to the pandemic, has been discussed as a crucial point for public health (10). Generally, stressful and uncertain situations increase anxiety and emphasize the individuals' need to receive social support by sharing similar experiences with others (11). Indeed, as previously stated, the loss of one's usual routine and reduced social contacts may cause frustration and a sense of isolation, which can generate high levels of distress (12–14). A 2-month follow-up study among Italian people during the Covid-19 lockdown showed an increase in stress and depression in the course of the lockdown (15). Relevant to the current study, this recent research has also shown that fewer coping strategies were associated with increased depression at follow-up. This suggests that how individuals dealt with their experience of isolation, including their need to communicate, belong to, and be part of a community, may well represent key issues during the COVID-19 pandemic.

Within this context, the use of social networking sites (SNSs) fulfilled the essential function of connection (16) by helping individuals to grow their social capital, and supporting relational closeness to the others *via* online interactions (17–20). The positive effects of SNSs have been clearly demonstrated, as they may promote positive functioning and foster positive emotional states (21, 22). Indeed, SNSs have been proposed as tools for alleviating anxiety during the COVID-19 pandemic (16), by allowing individuals to feel that they are not alone but part of a community (23). Smartphone apps and social technologies have had the potential to enhance individuals' experience of connectedness, despite the disclosed risks of infodemic and technological exhaustion (24–26). Accordingly, the positive central role of a recreational and needful use of videogames and SNSs in times of physical and social distancing, has been evidenced even though carefully addressed (27, 28), also suggesting that an excessive use of SNSs might temporarily act as a coping strategy (29, 30). However, some authors have recently argued that this coping mechanism might potentially lead to a longer-lasting threat (i.e., Problematic Social Networking Sites Use; PSNSU) in keeping with findings from a few recent studies (31, 32).

### Fear of Missing Out and Social Networking Site Use

The Fear of Missing Out (FoMO) is defined as “a pervasive apprehension that others might be having rewarding experiences from which one is absent, FoMO is characterized by the desire to stay continually connected with what others are doing. For those who fear missing out, participation in social media may be especially attractive” (31, p. 1841). Indeed, the online environment constitutes an ideal context to fulfill the need to be connected with the others and to be socially informed despite the distance, satisfying individuals' need for relatedness (10). For this reason, some studies [e.g., (33)] have focused their attention on the association between FoMO and Internet addiction. However, Internet addiction has been criticized as being an inadequate umbrella term that overlooks important differences between various online activities (34, 35) which, conversely, warrant specific and differentiated attention (36–38). Specifically, PSNSU has been defined as “being overly concerned about social networking sites (SNSs), to be driven by a strong motivation to log on to or use SNSs, and to devote so much time and effort to SNSs that it impairs other social activities, studies/job, interpersonal relationships, and/or psychological health and well-being” [(39), p. 4054]. Previous research has found a positive association between FoMO and social media misuse (40–43). Moreover, findings on gender-related differences suggested that women tend to score higher on FoMO than men (44, 45).

As the desire—or the need—to be continually connected with others is easily satisfied by using SNSs, it has been suggested that FoMO might be a risk factor for PSNSU. FoMO is a direct predictor of PSNSU use or a mediator in the relationships between psychopathological symptoms and negative outcomes arising from SNS use (46, 47). FoMO was also found to predict metacognitions associated with social media use, which, in turn, predict unregulated social media use (48). Thus, individuals may try to regulate their FoMO through massive use of social media because they believe that this tool is useful for regulating their fear of being excluded.

As pandemic does not constitute a usual life-circumstance, and social restrictions due to the COVID-19 epidemic have reduced the opportunities for social encounters, FoMO needs to be carefully questioned. Casale and Flett (10) have recently discussed the utility of the FoMO construct during the current pandemic, suggesting that this construct might become less relevant and salient because of the currently prevailing conditions. It might be the case that aspects of the psychological reality that this construct is intended to represent are either missing or have been drastically reduced. The FoMO construct includes, by definition, the possibility for significant others to have fun or to enjoy rewarding experiences, planning get-togethers, and meet up with friends. However, social isolation restricts the range of what friends are actually doing because their behavior is severely constrained. One might argue that if FoMO levels decrease in times of pandemic, unhealthy behaviors and negative outcomes related to high levels of FoMO (i.e., PSNUS) should show a decrease as well (10). Consequently, there is a need to investigate if the well-established positive association between FoMO levels and PSNUS remains stable during the pandemic or, instead, if it might be the case that PSNSU is driven by different psychological risk factors depending on the circumstances. Recent findings have reported that the psychological burden of the COVID-19 pandemic includes increased social media use in order to maintain social relationships (49). Individuals who are afraid of being invisible in the world of social media (8) and who are in situations of physical isolation will more likely need to find ways to stay connected with other people. Hence, these conditions might enhance massive or problematic SNSs use. Below we will describe the specific mechanisms that might explain how FoMO might impact on PSNSU in time of physical distancing.

### Online Communication Attitude and Relational Closeness Across Social Networking Sites

Computer-mediated communication (CMC) has been described as a digitally-mediated pattern of communication (50–52). For younger generations, CMC is essential to the initiation, development, and maintenance of interpersonal relationships (53). Within this context, the Online Communication Attitude (OCA) has been conceptualized as a cluster of cognitive and affective orientations, that is a trait-like attitude and relatively enduring organization of beliefs that leads individuals to respond in some preferential manner toward online communication, thus influencing online behaviors and relational outcomes (54, 55). More specifically, attitudes toward *online self-disclosure* (OSD) and *online social connection* (OSC) have been stated as two core features of individuals' OCA, affecting media-use patterns in the interpersonal relationship (54, 56). According to Ledbetter (54), those with a high attitude toward OSD feel more comfortable and less embarrassed when sharing personal information across social media and are less shy when communicating online, whereas those with high attitude toward OSC share the belief that loss of online communication would reduce contact with others and dramatically change their social life. It seems that attitudinal variables strongly predict the motives for socialization and interpersonal relationships development/maintenance *via* SNSs (57). In this regard, previous research has posited that the more people are prone to communicate *via* online social platforms (i.e., keeping social contacts and self-disclosing online), the more this attitude will influence their engagement in SNSs for interpersonal relationships and, in turn, relational closeness to friends across SNSs (54, 55).

In this regard, Vangelisti and Caughlin (58) highlighted the importance of psychological closeness to others within the context of personal disclosure. Later, according to Aron et al. (59), Ledbetter et al. (55) conceptualized *relational closeness* as “a subjective experience of intimacy, emotional affinity, and psychological bonding with another person” (p. 34), which plays a critical role in online relationships contributing to individuals' experiences of intimacy and emotional closeness. Moreover, assuming that self-disclosure and social connection are basic motivations that promote online interpersonal communication (54), it has been demonstrated that these attitudes toward online communication may directly influence relational closeness to the others *via* online relationships (55).

Therefore, relational closeness has been posited as an important interpersonal outcome, associated with online communication, supporting the dominance of close ties in the provision of social support *via* social media (60, 61). Similarly, comments from relationally close individuals are more supportive if compared to a relationally non-close reply (62, 63) and may influence adolescents' identity development, including sociability and self-esteem (64). In this regard, psychological outcomes should be considered depending on the healthy or unhealthy use of online communication and relationships. Accordingly, Baym and Ledbetter (65) already posited a strict association between the quality of relationship with SNS friends and the frequency of SNS contacts, as well as scientific research has increasingly explored the strong relationship between Internet use/misuse and interpersonal facets of Internet applications [e.g., (42, 66–71)].

In fact, the use of SNSs provides for social connections, information, and emotional content-sharing, as well as for experiences of online self-disclosure, intimacy, and emotional closeness. However, contradictory results concerning the use of new communication technologies highlighted positive (54, 72, 73) rather than deleterious (74–76) effects on the quality of interpersonal relationships. Specifically, despite online communication may fulfill critical needs of social interactions, self-disclosure, and identity exploration in young people (77), this attitude has been associated with compulsive Internet use and a specific preference for online social interactions (56). Moreover, even though responding to the need of facing negative emotions and searching for social support (31, 78, 79), the preference for computer-mediated interactions may trigger risky psychosocial and relational outcomes (80–82). Particularly, attitude toward OSC has emerged as a significant positive predictor of social media use (83) and relational closeness across SNSs (55), likely a healthy, communicatively competent motivation for using online communication. Conversely, OSD has been associated with negative psychosocial and relational outcomes, probably due to the individual's desire for over-controlling or falsifying personal self-presentation (55, 56, 80, 81). Accordingly, a recent study from the Authors (blinded reference for peer review), confirmed the association between OSD and negative relational outcomes suggesting that young adults who were prone to self-disclose online largely tend to prefer online social interactions. Moreover, these recent findings also reinforced previous few evidence on the predicting role that online communication attitudes may have on relational closeness with online friends (55).

Finally, gender-related differences have been indicated in individuals use of social media, thus showing that females disclose more than their male peers principally using social media for relational purposes (84–87). However, recently higher scores in men's self-disclosure and relational closeness with online friends (Authors, submitted, blinded reference) suggested a reconsideration of gender-related differences in online communication attitudes and social media use, addressing for further investigation.

Interestingly, the potential effect of FoMO on PSNSU through online self-disclosure and online social connection has not yet been the focus of scientific attention. On the one hand, previous studies supported a positive association between FoMO levels and problematic social media use. On the other hand, previous findings show that attitudes toward online communication directly predict relational closeness toward online friends and that the higher the attitude toward online self-disclosure and online social connection, the higher the compulsive use of social media. It is psychologically plausible that those who fear to be excluded might develop stronger attitudes toward online self-disclosure and online social connection in a time of physical and social distancing, in order to meet their need to be socially connected. That is, we speculated that in time of social restrictions, attitudes toward the online environment are enhanced because the forced lockdown might have merely transferred social interactions to the online environment and this, in turn, might put a person at risk to develop PSNSU.

### The Present Study

**Figure 1:**
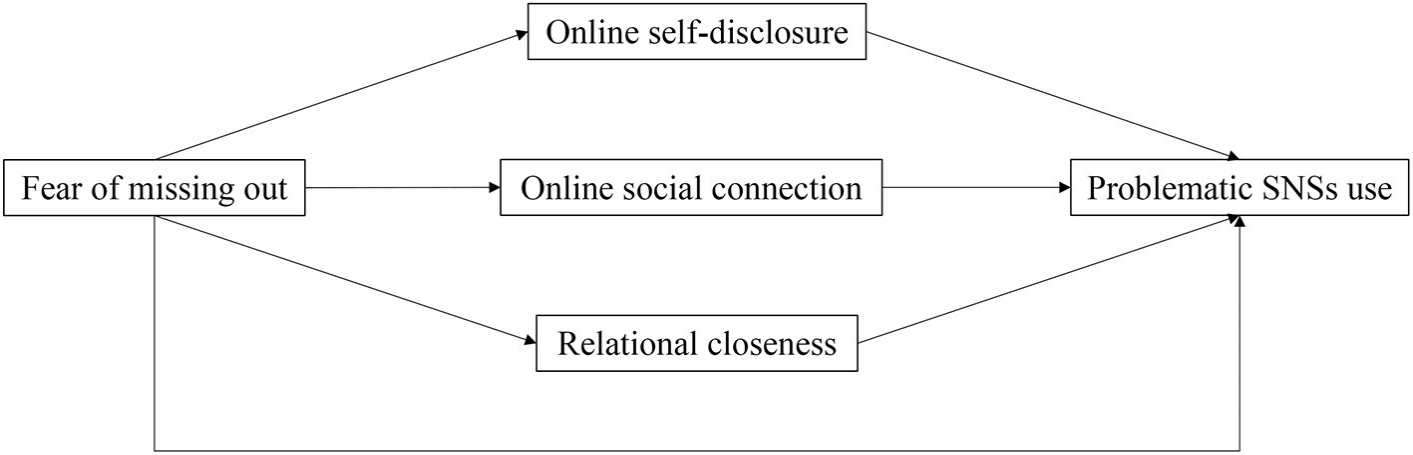
Hypothesized parallel mediation model.

Accordingly, we hypothesized that individuals who are afraid of being excluded or invisible to the others, in situations of physical isolation would more likely need to find ways to be close and connected, *to become visible* self-disclosing in the only possible context of interaction they could use during the pandemic lockdown. Therefore, the current study aimed to explore the predicting role of FoMO on PSNSU during the COVID-19 social restrictions, testing the mediating effect of the online communication attitude and online relational closeness on this relationship. In detail, we expected to find an association between FoMO levels and PSNSU in accordance with previous studies [e.g., (33, 41, 43, 46)]. Moreover, we expected that FoMO would influence the tendency toward online social connections and promote the need for interpersonal contacts and relational closeness to the others *via* online social interactions, which would lead in turn to PSNSU (Figure 1). Finally, since there are gender-related differences in individuals' attitude toward online communication and FoMO levels, we explored gender differences in this relationship.

Thus, the following hypotheses were proposed and tested:

H1: There will be self-reported higher use of SNS during the pandemic compared to previous levels;

H2: FoMO will positively affect PSNSU through online communication and relational closeness on social networking sites. We expected this mediation to be partial rather than full, as other mechanisms through which FoMO influences PSNSU (e.g., metacognitions) are also likely to operate.

## Methods

### Participants and Procedure

A total of 487 Italian adults responded to an online survey. The sample comprised 198 men (40.7%) and 289 women (59.3%) aged between 18 and 70 years, with a mean age of 29.85 years (SD = 9.76). Participants were recruited during the COVID-19 pandemic lockdown phase in Italy (specifically from April 1st to 30th 2020) *via* advertisements in Italian university Web communities and other online groups (*via* social media platforms), which asked for dissemination among their members. Therefore, a snowball sampling method was adopted as a recruitment strategy. The call for participation in the online study contained a website link for participants to click on in order to fill out the questionnaire. Participants were informed of the research aims, its scope, and the measures to be used in generating the data. Participation was voluntary. Confidentiality and anonymity were guaranteed. The participants could withdraw from the study at any time. No course credits or payment was given. There were no specific inclusion criteria, except that of being of legal age which, according to Italian law, is 18 years of age. The study was approved by the Research Ethics Committee of the University of Naples Federico II and was conducted according to the ethical guidelines for psychological research established by the Italian Psychological Association (AIP).

### Measures

#### Sociodemographic Information and Social Media Use Patterns

Information was collected about gender, age, ethnic origin, being student, marital status, geographical provenance, whether the participant was living alone during the quarantine, the most used social networking sites, and hours per day spent social networking before and during forced isolation due to COVID-19. A score was calculated that reflected the difference between the number of hours participants declared they spent on SNSs during and before the COVID-19 lockdown.

#### Fear of Missing Out Scale

The Italian version of the FoMO scale [(48); original English version by (88)] was used to evaluate the fears, worries, and anxiety people might have in relation to being out of touch with events, experiences, and conversations among their social circles (e.g., “*I fear my friends have more rewarding experiences than me*”). FoMO is a 10-item scale rated on a 5-point Likert scale ranging from 1 (*not at all true of me*) to 5 (*extremely true of me*). Higher scores indicate a higher Fear of Missing Out. The Cronbach alpha in the current study was α = 0.83.

#### Online Communication Attitude Scale

The online self-disclosure (OSD) and online social connection (OSC) subscales of the Italian version of the OCA scale [(54); Authors, submitted, blinded reference] were used. The online self-disclosure attitude subscale contains seven items (e.g., “*I feel like I can be more open when I am communicating online*”), and the online social connection subscale contains six items (e.g., “*I would communicate less with my friends if I couldn't talk with them online*”). Participants responded on a 7-point Likert-type scale with response options ranging from 1 (*strongly disagree*) to 7 (*strongly agree*). Cronbach's α values for the online self-disclosure and online social connection subscales were 0.91 and 0.82, respectively.

#### Relational Closeness

A preliminary Italian version of Vangelisti and Caughlin's (58) seven-item measure was used to assess relational closeness with online friends (e.g., “*How often do you talk about personal things with your online friends*?” and “*How close are you to your online friends?*”). Participants responded on a 7-point Likert-type scale ranging from 1 (*not at all*) to 7 (*very much*). The measure demonstrated strong internal reliability (α = 0.91).

This preliminary Italian version of the relational closeness measure was obtained using a back-translation method in which one translator translated the tests from the source language (English) to the target language (Italian). A second translator, without having seen the original test, translated the new versions of the tests back to the source language. The original and the back-translated versions of the tests were then compared, and judgments were made about their equivalence. Although not yet validated, this measure has been used in a previous study on Italian sample of adolescents and adults, showing a good internal consistency (α = 0.92) and a strong correlation with OCA and preference for online social interactions (POSI) [(89) unpublished thesis dissertation; Authors, submitted, blinded reference].

#### Generalized Problematic Internet Use Scale 2

The 15-item Italian version of GPIUS2 [(90) original English version by (91)] assesses the degree to which someone experiences the cognitions, behaviors, and outcomes arising because of the unique communicative context of the Internet on a scale ranging from 1 (*strongly disagree*) to 8 (*strongly agree*). Participants' scores on the 15 items can be added up to create an overall GPIU score. As in various previous studies (92, 93), since the GPIUS2 items are referred to the use of the Internet without differentiating between different activities carried out online, for the purposes of the present study the word “Internet” has been replaced by “social networking sites” (e.g., “*I have used SNS to feel better when I was down*”). In the current study, Cronbach's α was 0.90.

The online survey was administered to a pilot sample of 10 undergraduate volunteers (four men and six women), in order to explore possible difficulties with the items and the online survey.

### Statistical Analyses

Descriptive statistics were performed using the Statistical Package for Social Sciences SPSS (Version 23 for Windows) and it was used to assess the means, standard deviation of the variables, and confidence interval of means (CI: 95%). Independent *t*-tests were used to assess gender differences, and the magnitude of the differences was evaluated with effect sizes (Cohen's *d*). Pearson's correlations between the study variables were performed. A parallel mediation analysis was conducted by using Model 4 of Hayes's (94) Process Macro for SPSS to explore the mediating effect of online communication attitude and relational closeness between the fear of missing out and the problematic SNSs use. The bootstrapping method was used to produce 95% bias-corrected confidence intervals (CI) for the magnitude of these effects based on 1,000 resamples of the data. Based on previous studies (46, 47) we expected the magnitude for the direct effect between FoMO and PSNSU to be medium in effect size. With α = 0.05 and power = 0.80, a sample size to detect a correlation of 0.30 is N = 115 (one-tailed). For the indirect (mediated) effects, we considered empirically-based estimates of sample-sizes needed to detect a mediated effect, presented in Fritz and McKinnon [(95), **Table 3**]. Assuming direct path coefficients of β = 0.26 and using a bias-corrected bootstrapping method, the estimated sample size to detect a mediated effect with α = 0.05 and power = 0.80 is estimated to be N = 148. Thus, we deem our collected sample to be sufficient to detect the predicted effects.

## Results

### Descriptive Statistics and Bivariate Correlations

Among the participants, 100% were Caucasian, 37.6% were single and only 4.3% were living alone during the quarantine. Concerning the geographical provenance, 61.9% were from Southern Italy, 23.8% were from Northern Italy, 12.9% were from Central Italy, and 1.4% were from Italian islands. The most used social media were WhatsApp (96.9%), Facebook (85%), Instagram (76.6%), Facebook Messenger (53.6%), and Twitter (16.2%). Before the forced isolation due to COVID-19, 35.3% of the participants reported that they spent 1–2 h/day on SNSs, and only 12.1% spent more than 4 h/day. During the quarantine, the percentage corresponding to 1–2 h/day significantly decreased to 15.4%, and 36.4% of the participants declared that they spent more than 4 h/day social networking [$\chi_{\left(25\right)}^{2}$ = 449.16; *p* < 0.001; phi = 0.96].

Descriptive statistics and gender differences are reported in Table 1. No gender-related statistically significant differences have been found except in h/day spent on SNSs during the COVID-19 pandemic, with women obtaining higher mean scores than men with a moderate effect size.

**Table 1:** Means, standard deviations (SD), *t*-test, effects sizes (Cohen's *d*) for both genders, and confidence intervals (CI).

	**Total sample**	**Males**	**Females**			
	**Mean (SD)**	**Mean (SD)**	**Mean (SD)**	**t**	***d***	**95% CI**
Hour/day spent on SNSs before the COVID-19 pandemic	2.92 (1.321)	2.82 (1.394)	2.99 (1.267)	1.395	0.13	−0.069;0.409
Hour/day spent on SNSs during the COVID-19 pandemic	3.95 (1.467)	3.65 (1.472)	4.15 (1.432)	3.721^***^	0.34	0.235;0.760
Fear of missing out	2.346 (0.743)	2.28 (0.756)	2.387 (0.734)	1.509	0.14	−0.033;0.248
OCA self-disclosure	2.443 (1.33)	2.565 (1.403)	2.366 (1.278)	−1.559^.^	0.14	−0.45;0.052
OCA social connection	3.641 (1.366)	3.653 (1.413)	3.634 (1.338)	−0.140	0.01	−0.277;0.24
Relational closeness	4.404 (1.271)	4.347 (1.252)	4.439 (1.284)	0.758^.^	0.07	−0.148;0.333
Problematic SNS use	2.535 (1.107)	2.566 (1.153)	2.515 (1.078)	−0.480	0.04	−0.26;0.158

OCA, Online Communication Attitude.

*** *p < 0.001*.

Bivariate correlations between all variables are shown in Table 2. Overall, statistically significant positive correlations were found among Fear of Missing Out, online communication attitudes (i.e., online self-disclosure and online social connection), relational closeness, and problematic social networking sites use, as expected. Moreover, the higher the Fear of Missing Out levels, the higher the hours per day on SNSs during COVID-19 pandemic and problematic social networking sites use.

**Table 2:** Bivariate correlations between all variables estimated with 1,000 bootstrap sample.

	**1**	**2**	**3**	**4**	**5**	**6**	**7**	**8**	**9**	**10**
1. Gender	–									
2. Age	0.02	–								
3. Marital status	−0.23^***^	0.20^***^	–							
4. Living alone during COVID-19	−0.09^*^	−0.18^***^	0.11^*^	–						
5. Hours/day on SNSs during COVID-19 pandemic	−0.17^***^	−0.23^***^	−0.10^*^	0.05	–					
6. Fear of missing out	−0.07	−0.39^***^	−0.17^***^	0.07	0.22^***^	–				
7. OCA self-disclosure	0.07	−0.21^***^	−0.18^***^	−0.07	0.22^***^	0.41^***^	–			
8. OCA social connection	0.01	−0.30^***^	−0.09	0.01	0.24^***^	0.42^***^	0.49^***^	–		
9. relational closeness	−0.04	−0.17^***^	−0.10^*^	0.11^*^	0.21^***^	0.25^***^	0.14^**^	0.25^***^	–	
10. Problematic SNS use	0.02	−0.27^***^	−0.18^***^	<0.00	0.39^***^	0.52^***^	0.61^***^	0.59^***^	0.21^***^	–

*OCA, Online Communication Attitude*.

* p < 0.05;

** p < 0.01;

*** *p < 0.001*.

### Parallel Mediation Analysis

In order to test the direct and indirect effect of Fear of Missing Out on problematic social networking sites use *via* the online communication attitude and relational closeness, a parallel mediational analysis was conducted. As shown in Table 3, after controlling for participants' gender (females coded as 0, males coded as 1; β = 0.036; *p* = 0.64, ns), age (β = 0.001; *p* = 0.87, ns), marital status (single coded as 0, in a relationship coded as 1; β = −0.106; *p* = 0.18, ns), living alone during the quarantine (no coded as 0, yes coded as 1; β = 0.124; *p* = 0.53, ns), and the difference between h/day spent on SNSs during and before the COVID-19 pandemic (β = 0.104; *p* < 0.05), the Fear of Missing Out had a significant direct effect on online self-disclosure (*t* = 8.208; *p* < 0.001), online social connection (*t* = 7.9; *p* < 0.001), and relational closeness (*t* = 4.188; *p* < 0.001). Moreover, self-disclosure and social connection had a significant direct effect on problematic SNSs use (*t* = 9.39; *p* < 0.001 and *t* = 7.842; *p* < 0.001, respectively), whereas relational closeness did not show a significant effect (*t* = 0.391; *p* = 0.70). Finally, the positive and significant direct effect of fear of missing out on problematic social networking (*t* = 5.943; *p* < 0.001) increased in magnitude when mediators were included in the model (*t* = 11.13; *p* < 0.001). Analysis of the bias-corrected confidence intervals of the indirect effect of Fear of Missing Out on problematic SNSs use in the bootstrapped samples further revealed that the indirect effects *via* self-disclosure and social connection were significant. The total model accounted for a significant amount of variance in participants' problematic social networking [*R*^2^ = 0.54; *F*_(9, 447)_ = 58.285, *p* < 0.001].

**Table 3:** Direct and indirect effect of the fear of missing out on problematic SNS use via online communication attitudes and relational closeness.

			**BCa 95% CI**	
	**Coeff**.	**SE**	**Lower**	**Upper**
*Path estimates*				
Gender (male)	0.036	0.077	−0.115	0.187
Age	0.001	0.004	−0.008	0.009
Marital status (in relationship)	−0.106	0.079	−0.262	0.049
Living alone during the quarantine	0.124	0.196	−0.261	0.508
Difference between h/day on SNSs during and before the COVID-19	0.104^**^	0.034	0.037	0.172
FoMO → Online self-disclosure	0.682^***^	0.083	0.519	0.845
FoMO → Online social connection	0.671^***^	0.085	0.504	0.838
FoMO → Relational closeness	0.356^***^	0.085	0.189	0.523
Online self-disclosure → PSNSU	0.301^***^	0.032	0.238	0.365
Online social connection → PSNSU	0.249^***^	0.032	0.186	0.311
Relational closeness → PSNSU	0.012^n.s.^	0.03	−0.047	0.07
*Total effect*: FoMO → PSNSU	0.723^***^	0.065	0.595	0.850
FoMO → PSNSU	0.346^***^	0.058	0.232	0.460
			**BCa 95% CI**	
	**Effect**	**SE**	**Lower**	**Upper**
*Indirect effects*				
Total	0.377	0.047	0.291	0.473
M1	0.206	0.038	0.138	0.284
M2	0.167	0.029	0.114	0.228
M3	0.004	0.011	−0.016	0.028

*FoMO, Fear of Missing Out; PSNSU, Problematic Social Networking Sites Use; M1, Online self-disclosure; M2, Online social connection; M3, Relational closeness*.

** p < 0.01;

*** *p < 0.001. n.s., non-significant*.

## Discussion

The present study aimed to explore the direct and indirect effect of FoMO on problematic SNS use *via* individuals' online communication attitude and relational closeness in a sample of Italian adults during the COVID-19 lockdown phase. It has been hypothesized that the use of SNSs would have been grown in this specific circumstance in order to preserve social connections and that FoMO would have been acted as a predictor of PSNSU. Moreover, we hypothesized that this predictive role would have been mediated by the attitude toward online self-disclosure and social connection and by the effect of the relational closeness to others *via* social interactions. Our results only partially confirmed these hypotheses. As expected, participants declared they spent more time on SNSs during the pandemic, particularly women, thus supporting previous results showing an increase in the hours per day spent using social media during the pandemic (31). Furthermore, our findings are aligned with all the previous results concerning the association between FoMO and PSNSU [e.g., (41, 46)] as FoMO directly predicted PSNSU. However, our results also built upon these previous studies as they highlight that the association remains stable in a period when one of the aspects of the psychological reality that this construct represents (i.e., others' socially rewarding experiences) has been drastically reduced because of the lockdown. The fear of being excluded from what's going on “outside” might have been transferred to what's going on “at home,” in the experience of online social encounters among friends that constituted the only chance to socialize that people had during the pandemic isolation. Moreover, the need for “ego validation” through comparison, which usually underlies individuals' use of social media and their fear of being excluded, might have been high, despite the social restrictions that limited people's behaviors inside their homes. Indeed, comparison, emotional sharing and social encounters have probably been addressed toward what friends were doing at home. Furthermore, we hypothesized that online communication attitudes (i.e., attitudes toward online social connectiveness and self-disclosure as well as the need of relational closeness *via* online interactions) would have been influenced by the experience of FoMO from the online environment, in times of offline social restrictions, consequently promoting a problematic use of SNSs. Our findings confirmed our hypothesis highlighting the influence that OCA—and particularly online self-disclosure—has in predicting the problematic use of SNSs under these circumstances. The predictive role of online communication attitudes with respect to problematic Internet use had already been highlighted by a previous study (56), but it has not been previously investigated in the context of social media use. Therefore, the current study builds upon previous results by showing that individuals' attitudes to online self-disclosure and social connection is involved in the link between FoMO and PSNSU. Conversely, and unexpectedly, relational closeness across SNSs did not predict individuals' use of SNS. We hypothesized that, during the COVID-19-induced social restrictions, individuals' use of social media would have been increased also because of the need to feel close to friends *via* online social connections, experiencing emotional closeness to the others and searching for support from close ties (62, 63) during these difficult circumstances. However, the present findings showed the pivotal role of attitudinal variables toward online communication, which seem to strongly predict individuals' development/maintenance of interpersonal relationships *via* SNSs (57), whereas we did not find a support for the role of relational closeness. Moreover, people's increase in SNS misuse seems to be a reaction to FoMO strengthened by individuals' trait-like attitudes toward online social connections and self-disclosure, more than by individuals' need to experience closeness to the others.

We can assume that the COVID-19 restrictions strengthened individual's use of social media and that those experiencing FoMO tried to regulate their fears by means a massive/problematic SNS use, improved by their preexisting attitudes toward online communication which might be reinforced, on their own, by this specific circumstance of social-distancing. These findings need to be addressed, as they seem to suggest that online communication trait-like attitudes might be a potential risk factor for social media misuse/abuse if linked to a real experience of social isolation and/or a fear of being deprived of the possibility of relatedness with others and of being involved in their experiences. However, further exploration on the association between FoMO and OCA are needed.

Moreover, although SNS use temporarily acted as a useful coping strategy with which to face social isolation (26, 29, 30), their massive use in this specific circumstance could have long-lasting effects on people with high levels of separation anxiety and fears of being excluded, and on those individuals who are more prone to using online communication strategies for connection and self-disclosure. Thus, longitudinal designs are greatly needed to analyze the pandemic's effects on social media use in different populations in greater depth, and the differences and similarities between different cultural contexts should be explored together with age and gender differences. The current study has some limitations that need to be addressed. First, the cross-sectional design limited the ability to formally test the causative effects. Second, the well-known risk of desirability biases due to the use of self-reported measures is also prevailing. Moreover, while considering the risks and the opportunities due to the online data collection (96), this study was conducted during the period of COVID-19 pandemic and specifically focused on individuals' behavior during the lockdown, thus online administration was the only possible and useful data collection among the population. Finally, since the current study refers to individuals' behavior across SNS during the COVID-19 epidemic, we cannot assume that their social media use would have been the same in different conditions. Therefore, our findings cannot be generalized. However, further research should investigate in-depth the influence that individuals' attitudes toward social connectiveness, self-disclosure and relational closeness across SNSs, could have on their use of social media in more regular circumstances. Indeed, within this context online relational closeness neither acted as a predictor nor as a mediator of problematic SNS use, although this feature is still little studied. Further research could explore the role that experiencing intimacy and emotional closeness across SNSs might have on a non-problematic use of social media, taking into account cultural, gender and age differences. Despite these limitations, the current findings have some theoretical and clinical implications. They built upon previous results regarding the effect of FoMO levels on PSNUS by showing that the usefulness of SNSs to regulate this specific fear remains stable during the experience of isolation and separation. Accordingly, this association between FoMO and PSNSU deserves clinical interest, especially considering the unexplored role of OCA in this relationship. Indeed, further exploration is needed on the role of online communication as a trait-like attitude potentially influencing individuals' unregulated use of social media. This study suggests deepening the risks related to the connection between the experience of FoMO and online self-disclosure and social connection. The fear to be excluded/invisible and the consistent urgency to become visible within the media environment should be carefully questioned also relating to identity developmental issues, as they both might widely affect online social encounters and promote dangerous individuals' hyper-self-disclosure or false self-presentation.

## Data Availability Statement

The raw data supporting the conclusions of this article will be made available by the authors, without undue reservation.

## Ethics Statement

The studies involving human participants were reviewed and approved by Ethical Committee of Psychological Research - Department of Humanities University of Naples Federico II. The patients/participants provided their written informed consent to participate in this study.

## Author Contributions

VB designed the study and contributed to writing a first draft of the manuscript. FG contributed to data collection, statistical analysis, and contributed to writing the first draft of the manuscript. GF contributed to developing methodology and statistical analysis. SC revised the whole work critically for important intellectual content. All authors read and approved the final version of the work.

## Conflict of Interest

The authors declare that the research was conducted in the absence of any commercial or financial relationships that could be construed as a potential conflict of interest.
